# Retraction statement: miR‐30d‐5p suppresses proliferation and autophagy by targeting ATG5 in renal cell carcinoma

**DOI:** 10.1002/2211-5463.13529

**Published:** 2022-12-12

https://doi.org/10.1002/2211-5463.13025

The above article published online on 03 November 2020 in Wiley Online Library (wileyonlinelibrary.com) has been retracted by agreement between the Editor‐in‐Chief, John Wiley and Sons Ltd, and the authors. The retraction has been agreed upon following an investigation based on allegations raised by a third party, which revealed inappropriate and unexplained duplications between this and a previously published article [1]. Thus, the editors consider the conclusions of this manuscript substantially compromised.

The first author of this article admitted to adding the corresponding author to this paper without their consent.
